# Lysophosphatidylcholines and Chlorophyll-Derived Molecules from the Diatom *Cylindrotheca closterium* with Anti-Inflammatory Activity

**DOI:** 10.3390/md18030166

**Published:** 2020-03-17

**Authors:** Chiara Lauritano, Kirsti Helland, Gennaro Riccio, Jeanette H. Andersen, Adrianna Ianora, Espen H. Hansen

**Affiliations:** 1 Department of Marine Biotechnology, Stazione Zoologica Anton Dohrn, CAP80121 Naples, Italy; gennaro.riccio@szn.it (G.R.); adrianna.ianora@szn.it (A.I.); 2 Marbio, UiT—The Arctic University of Norway, Breivika N-9037 Tromsø, Norway; kirsti.helland@uit.no (K.H.); jeanette.h.andersen@uit.no (J.H.A.); espen.hansen@uit.no (E.H.H.)

**Keywords:** diatoms, marine biotechnology, anti-inflammatory, drug discovery, *Cylindrotheca closterium*

## Abstract

Microalgae have been shown to be excellent producers of lipids, pigments, carbohydrates, and a plethora of secondary metabolites with possible applications in the pharmacological, nutraceutical, and cosmeceutical sectors. Recently, various microalgal raw extracts have been found to have anti-inflammatory properties. In this study, we performed the fractionation of raw extracts of the diatom *Cylindrotheca closterium,* previously shown to have anti-inflammatory properties, obtaining five fractions. Fractions C and D were found to significantly inhibit tumor necrosis factor alpha (TNF-⍺) release in LPS-stimulated human monocyte THP-1 cells. A dereplication analysis of these two fractions allowed the identification of their main components. Our data suggest that lysophosphatidylcholines and a breakdown product of chlorophyll, pheophorbide a, were probably responsible for the observed anti-inflammatory activity. Pheophorbide a is known to have anti-inflammatory properties. We tested and confirmed the anti-inflammatory activity of 1-palmitoyl-sn-glycero-3-phosphocholine, the most abundant lysophosphatidylcholine found in fraction C. This study demonstrated the importance of proper dereplication of bioactive extracts and fractions before isolation of compounds is commenced.

## 1. Introduction

Inflammation is a complex set of interactions among soluble factors and cells (e.g., chemokines, cytokines, adhesion molecules, recruitment, and activation of leukocytes) that can arise in any tissue helping to protect the host from systemic infection and to restore tissue homeostasis after injury, infection, and irritation [1,2,3]. Therefore, it represents a crucial defense mechanism that is important for maintenance of health [1,2]. However, if targeted destruction and assisted repair are not properly controlled by its mediators, the so-called “non-resolving inflammation”, they can lead to persistent tissue damage and the insurgence of various pathologies [2]. Inflammation has important pathogenic roles in several pathologies, such as asthma, atherosclerosis, atopic dermatitis, Crohn’s disease, multiple sclerosis, cystic fibrosis, psoriasis, neurodegenerative diseases, as well as cancer [1,4]. It is a protective response that involves immune cells, blood vessels, and different molecular mediators (e.g., TNF-α, IL1, nitric oxide, and prostaglandins) and anti-inflammatory assays that are used in the literature, generally include the study of one or more of these characteristics and mediators.

Oceans account for 71% of the earth’s surface and represent a huge, relatively untapped, reservoir of new compounds for drug discovery [5]. One such source is the Phytoplankton, photosynthetic eukaryotes at the base of marine and freshwater food webs that are essential in the transfer of organic material to top consumers such as fish [6]. These micro-organisms have shorter generation times as compared with macro-organisms and can easily be cultivated in closed photobioreactors or in open ponds providing access to larger amounts of biomass necessary for an eco-sustainable and eco-friendly approach to drug discovery [7]. Diatoms, with over 100,000 species, constitute one of the major components of marine phytoplankton, comprise up to 40% of annual productivity at sea [8] and represent 25% of global carbon-fixation [9]. Different studies have shown that diatoms are excellent sources and producers of pigments, lipids, and bioactive compounds [7,10]. Anti-inflammatory properties have previously been found for various diatoms, such as *Porosira glacialis*, *Attheya longicornis* [11], *Cylindrotheca closterium*, *Odontella mobiliensis*, *Pseudonitzschia pseudodelicatissima* [12], and *Phaeodactylum tricornutum* [13]. The activity was assessed on lipopolysaccaride (LPS)-stimulated human THP-1 macrophages, except for *P. tricornutum* which was tested on murine RAW 264.7 macrophages. However, there is very little information available on the actual compounds responsible for the observed anti-inflammatory activity. 

Anti-inflammatory properties have also been found for flagellates. Extracts of *Tetraselmis suecica* [14], *Chlorella ovalis*, *Nannochloropsis oculata,* and *Amphidinium carterae* [13], and a sterol-rich fraction of *Nannochloropsis oculata* [15] were active in LPS-stimulated RAW 264.7 macrophages. Oxylipin-containing lyophilized biomass from *Chlamydomonas debaryana* have been shown to have anti-inflammatory properties in an induced colitis rat model [16,17,18] and *Dunaliella bardawil* was found to protect against acetic acid-induced small bowel inflammation in rats [16,17]. Regarding studies on LPS-stimulated human THP-1 monocytic leukemia cells, lipid extracts of *Pavlova lutheri* [19] and monogalactosyldiacylglycerols (MGDGs) and digalactosyldiacylglycerols (DGDGs) mixtures, and the isolated DGDGs 11 and 12 from *Isochrysis galbana* [20] were reported as active. Regarding the compounds responsible for anti-inflammatory properties from flagellates, lycopene was purified from *Chlorella marina* and the activity was confirmed in a rat model of arthritis [21], and phytosterols from *Dunaliella tertiolecta* were tested in a sheep model of inflammation [22]. In addition, carotenoids, the most abundant lipid-soluble phytochemicals, have shown anti-inflammatory properties [23].

In this study, we investigated the capacity of extracts of *C. closterium* to inhibit the release of one of the main effectors of inflammation, TNF-α [3], in LPS-stimulated human THP-1 monocytic leukemia cells. Bioactivity-guided fractionation was performed, and chemical contents of the active fraction are described for the first time. This study is perfectly aligned with recent trends in analyzing possible microalgal properties for cancer prevention and improving general human health and well-being [24,25]. 

## 2. Results and Discussion

### 2.1. Testing for Anti-Inflammatory Activity in Algal Fractions

**Figure 1:**
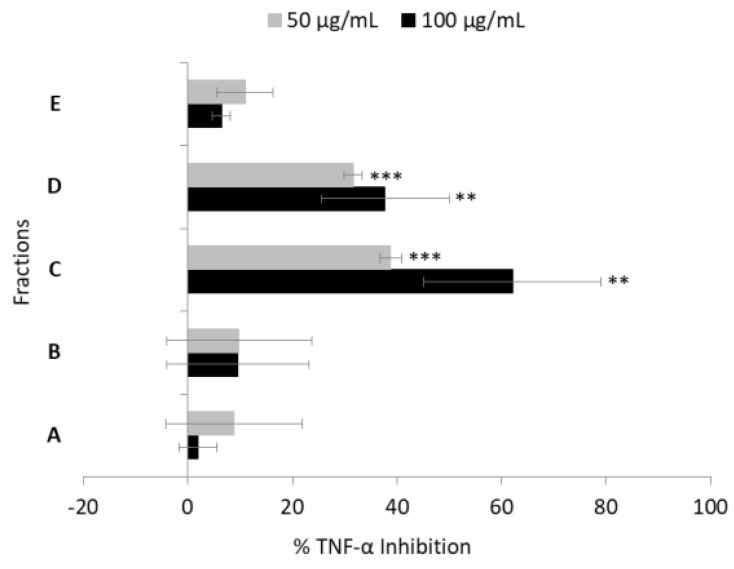
Anti-inflammatory assay. Inhibition of TNF-α secretion from LPS-stimulated THP-1 cells treated with fractions A, B, C, D, and E of *Cylindrotheca closterium* extracts (*n* = 3, ** for *p* < 0.01 and *** for *p* < 0.001, Student’s *t*-test).

Previous studies have shown that raw extracts of the diatom *C. closterium* had anti-inflammatory properties [12]. In the present study, a raw extract of *C. closterium* was pre-fractionated to obtain five fractions (Fractions A to E). These were amino acids and saccharides rich fraction (named Fraction A), nucleosides rich fraction (named fraction B), glycol- and phospholipid rich fraction (named fraction C), free fatty acids and sterols rich fraction (named fraction D), and triglycerides rich fraction (named fraction E), as reported in the solid phase extraction (SPE) protocol to fractionate organic extracts of Cutignano et al. [26]. Bioactivity testing of these fractions identified fraction C as the most active, able to inhibit TNF-α release at 100 µg/mL and 50 µg/mL concentrations (Figure 1). In particular, at 100 µg/mL, fraction C showed 60% inhibition of TNF-α release (*p* < 0.01), and 40% inhibition at 50 µg/mL (*p* < 0.001). Fraction D showed almost 40% TNF-α inhibition at 100 µg/mL (*p* < 0.01) and 30% at 50 µg/mL (*p* < 0.001). The other fractions did not show any significant TNF-α inhibition activity (*p* > 0.05). Both fractions C and D were selected for dereplication and further characterization.

### 2.2. Anti-Proliferative Activity Assay 

**Figure 2:**
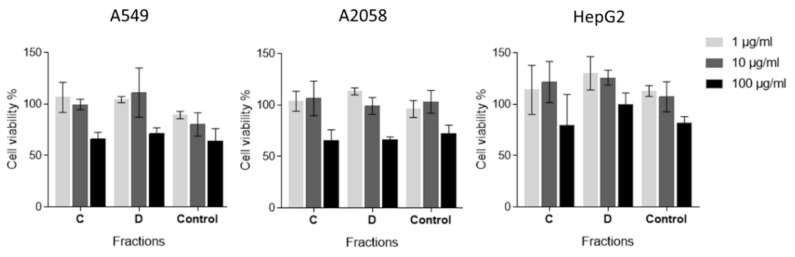
Antiproliferative assay. The histograms show the antiproliferative effects of fractions C and D of *C. closterium* extracts, on A549, A2058, and HepG2 cell lines. Control sample, containing only DMSO, was also tested (named as control). Results are expressed as percent survival after 72 h exposure (*n* = 3).

In order to test if the active anti-inflammatory fractions of *C. closterium* also had antiproliferative activities, the 3-(4,5-dimethyl-2-thizolyl)-2,5-diphenyl-2H-tetrazolium bromide (MTT) assay was performed. In particular, A549, A2058, and HepG2 cells were incubated in the presence or in the absence of three different concentrations (1, 10, and 100 μg/mL) of both fractions C and D. After 72 h of incubation at 37 °C, cell survival was measured with the MTT assay. As shown in Figure 2, fractions C and D did not show any significant inhibition of cell proliferation (*p* > 0.05). These results suggest that these two fractions have no antiproliferative or cytotoxicity activity but specific anti-inflammatory activity.

### 2.3. Dereplication

**Figure 3:**
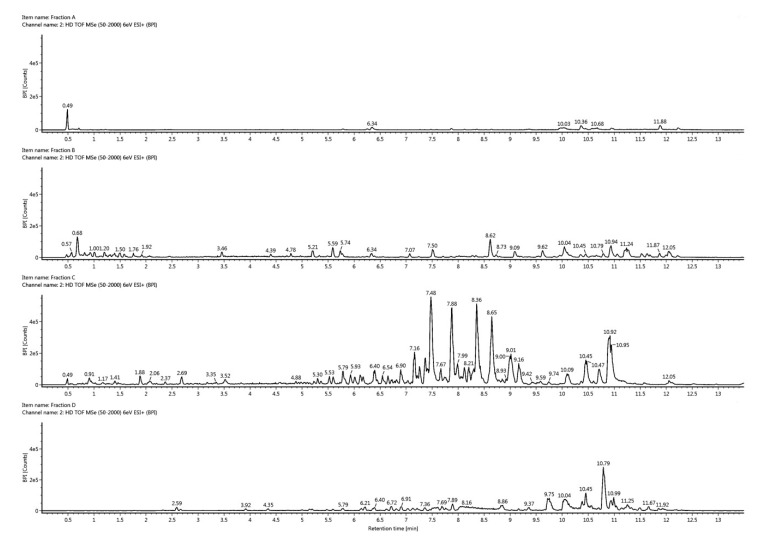
Base peak intensity chromatograms of fraction A, B, C, and D from the UHPLC-HR-MS/MS analysis using positive electrospray.

Since isolation of new compounds is very time consuming and costly [27,28], it is important to perform an early dereplication to identify already known components. In order to identify the bioactive compounds in the active fractions C and D, they were analyzed by UHPLC-HR-MS/MS and compared to the inactive fractions A and B (Figure 3). In fraction C, we found a series of compounds that all had a common fragment at *m/z* 184.0740 corresponding to a molecular formula of C_5_H_15_NO_4_P (see Figure S1, Supplementary Information). This is a common fragment observed when the head group of phosphocholines is cleaved off in tandem mass spectrometry. Phosphocholines are a class of phospholipids where the phosphocholine head group can be esterified to one or two fatty acids. Phosphocholines with two fatty acids are common membrane-forming phospholipids known as phosphatidylcholines (PC). When one fatty acid is removed from a PC, either enzymatically or by spontaneous hydrolysis, lysophosphatidylcholines (LysoPCs) are formed. After calculating the elemental compositions of the related molecules in fraction C and searching the ChemSpider database for known compounds, they were all identified as LysoPCs with different fatty acids attached. From the UHPLC-HR-MS/MS data we were able to determine the length of the fatty acids and the degree of unsaturation, but we were not able to directly determine the position of any double bonds and if the fatty acid was attached to carbon one or two on the glycerol backbone. In order to confirm our identification of LysoPCs, we injected a commercial standard of a C16:0 LysoPC (1-palmiotyl-sn-glycero-3-phosphocholine). The standard had the same retention time, mass, collisional cross section, and fragmentation pattern as one of the most intense compounds in fraction C (see Figure S2 and S3, Supplementary Information). The dominating LysoPCs in fraction C were 16:0, 16:1, 18:1, and 18:2 (approximately equal amounts) and minor LysoPCs were 14:0 and 18:3 (each approximately 20% of the most intense LysoPCs). A summary of the most intense LysoPCs and their retention times is given in Table S1 (Supplementary Information). There is current interest in LysoPCs because some of these are proposed for treatment of systemic inflammatory disorders [29,30,31,32,33,34,35]. However, their biological roles are not completely understood and some studies even found a putative pro-inflammatory activity [29]. Plasma LysoPC levels are diminished in human patients with sepsis [31,36], and in rodent models of sepsis and ischemia, LysoPC treatments in ex vivo and in vivo studies suggesting a potential role to relieve serious inflammatory conditions [29]. LysoPCs have also been shown to prevent neuronal death both in an in vivo model of transient global ischemia and in an in vitro model of excitotoxicity using primary cultures of cerebellar granule cells exposed to high extracellular concentrations of glutamate (20 to 40 micromol/L). 

In fraction D, trace amounts of the same LysoPCs were present. However, the most intense peak in fraction D had a *m/z* value of 593.2752 with a corresponding elemental composition of C_35_H_37_N_4_O_5_ ([M + H]^+^). When searching in the ChemSpider database, the elemental composition, as well as the fragmentation pattern, indicated that the compound was pheophorbide a, a breakdown product of chlorophyll (see Figure S4, Supplementary Information). Another peak in fraction D was identified as a related breakdown product of chlorophyll, hydroxypheophorbide a (C_35_H_36_N_4_O_6_, *m/z* 609.2708 [M + H]^+^), see Figure S5 (Supplementary Information). Both pheophorbide a and its derivatives are known to have anti-inflammatory and anticancer properties [37,38,39,40,41,42,43], but, to our knowledge, this is the first case in a marine microalgae where the bioactivity was attributed to pheophorbide a. Pheophorbide a has already been extracted from a range of different marine organisms. Examples are the seaweed *Grateloupia ellittica* [40], the brown alga *Saccharina japonica* [39], marine diatoms [44,45], and the freshwater glaucophyte *Cyanophora paradoxa* [37]. Hydroxypheophorbide a has been previously isolated from the terrestrial plants *Clerodendrum calamitosum*, *Neptunia oleracea*, the freshwater unicellular green alga *Chlorella* sp., and from the marine tunicate *Trididemnum solidum*, but never from a marine diatom species. It is mainly known to have anticancer but not anti-inflammatory activity ([38,41], Patents No. 185220/82 and US4709022A). Hence we suggest that the possible anti-inflammatory activity observed in our experiments was due to the presence of LysoPCs and the known anti-inflammatory pheophorbide a. In fact, pheophorbide a is known to induce a dose-dependent inhibition against lipopolysaccharide (LPS)-induced nitric oxide (NO) production at nontoxic concentrations in RAW 264.7 murine macrophage cells and to suppress the expression of nitric oxide synthase (iNOS) [39]. 

### 2.4. Anti-Inflammatory Activity of 1-Palmitoyl-sn-glycero-3-phosphocholine

**Figure 4:**
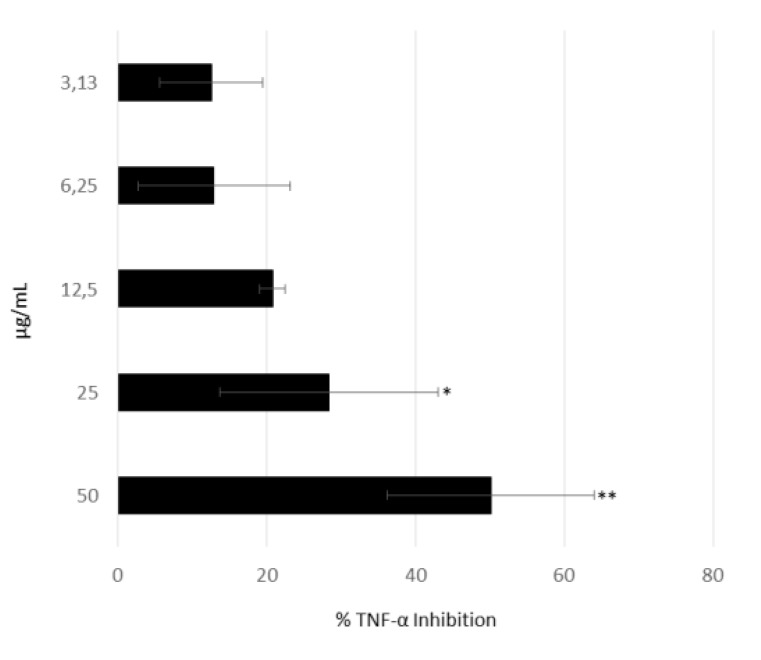
Anti-inflammatory assay. Inhibition of TNF-α secretion from LPS-stimulated THP-1 cells treated with 3.13, 6.25, 12.5, 25, and 50 μg/mL of 1-Palmitoyl-sn-glycero-3-phosphocholine (*n* = 3; * for *p* < 0.05 and ** for *p* < 0.01, Student’s *t*-test).

Considering that the most active fraction mainly contained various phosphocholines, we tested one of these, 1-palmitoyl-sn-glycero-3-phosphocholine (which was the most abundant compound in fraction C) in our AIF-assay. The effect of 1-palmitoyl-sn-glycero-3-phosphocholine on secretion of TNF-⍺ showed a dose-response relationship and was active at 25 µg/mL (*p* < 0.05) and 50 µg/mL (*p* < 0.01), as shown in Figure 4. 

## 3. Conclusions

Considering that inflammation plays a crucial role in the pathogenicity of several diseases, marine drug discovery is often directed to finding new natural products with anti-inflammatory properties [46,47]. Our results indicate that lysophosphatidylcholines (lysoPCs) and a breakdown product of chlorophyll, pheophorbide a, were probably responsible for the observed anti-inflammatory activity of the diatom *C. closterium*, giving new insights into microalgal compound bioactivities and their possible applications. This is the first time that a marine diatom is reported to produce these anti-inflammatory compounds. Pheophorbide a is known to inhibit the production of NO via inhibition of iNOS protein expression, thereby suggesting its potential use in the treatment of various inflammatory diseases (37), but there is no further information on its anti-inflammatory mechanism of action. Hence our study shows, for the first time, that it can inhibit TNF-α release in THP1 cells. 

LysoPCs are products of phospholipase A2 enzyme activity, and similar to the enzyme, have a direct role in pro-inflammatory [48] and anti-inflammatory responses, in a variety of organ systems. Our results indicate that one of these LysoPCs, 1-palmitoyl-sn-glycero-3-phosphocholine (which was the most abundant compound in fraction C), had a strong anti-inflammatory activity which has not been demonstrated before. Microalgae, and in particular diatoms, therefore, can be considered potentially important producers of compounds to prevent and treat different human pathologies. In recent years they have been shown to have anti-inflammatory, antimicrobial, anticancer, antidiabetic, antiepileptic and even antituberculosis properties [10,11,12,49,50,51,52,53,54,55]. A better understanding of the potential health benefits from these marine organisms, the compounds they produce, and the environmental conditions affecting their production should allow for the sustainable development of these valuable marine resources in the future.

## 4. Materials and Methods 

### 4.1. Cell Culturing and Harvesting

The diatom *Cylindrotheca closterium* (FE2), which has previously been shown to have anti-inflammatory activity [12], was cultured in Guillard’s f/2 medium [56] in ten-liter polycarbonate carboys (4 replicates). Cultures were constantly bubbled with air filtered through 0.2 μm membrane filters and kept in a climate chamber at 19 °C and a 12:12 h light:dark cycle (100 μmol photons m^−2^ s^−1^). Initial cell concentration was about 5000 cells/mL per bottle; culture growth was monitored daily by fixing 1 ml of culture with one drop of Lugol (final concentration of about 2%) and counting cells in a Bürker counting chamber under an Axioskop 2 microscope (20×) (Carl Zeiss GmbH, Jena, Germany). At the end of the stationary phase, cultures were centrifuged for 15 min at 4 °C at 3900 g using a cooled centrifuge with a swing-out rotor (DR 15P, Braun Biotechnology International, Allentown, PA, USA). The supernatant was discarded, and pellets freeze-dried and kept at −80 °C until chemical extraction. 

### 4.2. Extraction and Fractionation

For extraction and fractionation, the protocol by Cutignano et al. [26] was used, but with some modifications. Briefly, a methanolic extract was firstly prepared by adding 5 mL methanol (MeOH) for each g of pellet (algae were cultured in three different occasions in triplicate batches), sonicating the samples for 30 s, centrifuging them at 4000 rpm for 5 min at room temperature and drying the supernatant with a rotovapor.

For the fractionation, columns were activated (column type: 6 mL/500 mg resin) with 6 mL methanol and 17 mL of distilled water. The resin used was a spherical poly(styrene-divinylbenzene) resin for SPE (Chromabond® HR-X, Düren, Germany). One mL of distilled water was added for each 20 mg of methanolic extract. Samples were sonicated to obtain a good suspension and added to the columns. The column was eluted as follows: (1) wash step with 2 mL of distilled water, throwing away 1.5 mL to eliminate salts, (2) addition of 18 mL of distilled water to obtain fraction A, (3) addition of 24 mL of methanol (CH_3_OH)/water (50:50) to obtain fraction B, (4) addition of 18 mL Acetonitrile (CH_3_CN)/water (70:30) to obtain fraction C, (5) addition of 18 mL acetonitrile (100%) to obtain fraction D, (6) and finally addition of 18 mL of dichloromethane/methanol (CH_2_Cl_2_/CH_3_OH; 90:10) to obtain fraction E.

### 4.3. Anti-Inflammatory Assay

The anti-inflammatory assay was performed as in Lauritano et al. [12]. Briefly, ∼10^6^ human monocyte THP-1 cells/mL (ATCC® TIB-202^TM^) supplemented with 50 ng/mL phorbol 12- myristate 13-acetate (PMA, Sigma-Aldrich) were seeded in 96-well plates and incubated at 37 °C, 5% CO_2_ for 48 h in RPMI-1640 medium (Biochrom). After 72 h, 80 μL fresh RPMI medium and 10 μL/well of test sample were added. In particular, fractions A, B, C, D, and E were tested at 100 and 50 μg/mL, while 1-palmitoyl-sn-glycero-3-phosphocholine (Sigma L5254) was tested at 3.13, 6.25, 12.5, 25, and 50 μg/mL. The tests were performed at least in triplicate. After incubation for 1 h, all samples were incubated with 1 ng/mL lipopolysaccharide (LPS) for 6 h at 37 °C and plates were then frozen at −80 °C. Enzyme-linked immunosorbent assay (ELISA) was used to test TNF-α inhibition. ELISA was performed as in Lauritano et al. [12]. Results were read at 405 nm.

### 4.4. In Vitro Anti-Proliferative Assay

Human cells were bought at ATCC (https://www.lgcstandards-atcc.org/). Human hepatocellular liver carcinoma cells (HepG2; ATCC® HB-8065™) were cultured in EMEM medium, human melanoma cells (A2058; ATCC®CRL-11147^TM^) were cultured in DMEM, adenocarcinomic human alveolar basal epithelial cells (A549; ATCC®CL-185^TM^) were cultured in F-12K medium. The media were supplemented with 10% fetal bovine serum, 50 U/ml penicillin, and 50 μg/ml streptomycin.

To evaluate the in vitro antiproliferative effects of the fractions, HepG2, A2058, and A549 cell lines were seeded in 96-well microtiter plates at a density of 1 × 10^4^ cells/well and incubated at 37 °C to allow for cell adhesion in the plates. After 16 h, the medium was replaced with fresh medium containing increasing concentrations of the fractions (1, 10, and 100 μg/mL) for 72 h. Each concentration was tested at least in triplicate. After 72 h, cell viability was assessed using the MTT test (3-(4,5-dimethyl-2-thizolyl)-2,5-diphenyl-2H-tetrazolium bromide; A2231,0001, AppliChem Panreac Tischkalender, Darmstadt, GmbH). Briefly, the medium was replaced with medium containing MTT at 0.5 mg/ml and the plates were incubated for 3 h at 37 °C. After incubation, cells were treated with isopropyl alcohol (used as MTT solvent) for 30 minutes at room temperature. Absorbance was measured at OD = 570 nm using a microplate reader (Multiskan™ FC Microplate Photometer, Thermo Fisher Scientific, Waltham, MA, USA). Cell survival was expressed as a percentage of viable cells in the presence of the tested samples, with respect to untreated control cultures.

### 4.5. Statistical Analysis

Student’s t-test was performed using GraphPad Prism statistic software, V4.00 (GraphPad Software, San Diego, CA, USA). Data were considered significant when p values were <0.05 (∗ for *p* < 0.05, ∗∗ for *p* < 0.01, and ∗∗∗ for *p* < 0.001).

### 4.6. Dereplication of Fractions

One mg of each fraction A to E were resuspended in 100 µL 80% aqueous methanol, centrifuged at 13,000 rpm for 5 min, and the supernatants were transferred to UHPLC injection vials. UHPLC-HR-MS/MS analysis of the fractions was performed using a Waters Acquity I-class UPLC system interfaced with a PDA Detector and a VION IMS-qTOF (Milford, MA, USA) using electrospray ionization (ESI) in positive mode. The VION IMS- qTOF was operated with a capillary voltage of 0.80 kV, desolvation gas flow (N_2_) of 800 L/h, desolvation temperature of 450 °C, cone gas flow (N_2_) of 50 L/h and an ion source temperature of 120 °C. Data were acquired between *m/z* 50 and 2000 with a scan time of 0.2 s. Fragment data were acquired by ramping the energy of the collision cell from 15 to 45 V, and high and low energy data were acquired in the same run. Leucine-enkephalin was used for internal calibration and the system was tuned to a resolution of 45,000 (FWHM). The system was controlled, and data were processed using UNIFI 1.9.4 (Waters). Chromatographic separation was achieved by injecting 3 µL of the dissolved fractions on a BEH C18 1.7 µm (2.1 × 100 mm) column (Waters) operated at 40 °C. The fractions were eluted with a gradient of 10% to 100% acetonitrile in water over 10 min (both containing 0.1% formic acid), followed by maintaining 100% acetonitrile until 13.5 min.

## Supplementary Materials

The following are available online at https://www.mdpi.com/1660-3397/18/3/166/s1, Figure S1: Top: Base peak intensity chromatogram from the UHPLC-HR-MS/MS analysis of fraction C using positive electrospray. Bottom: Ion chromatogram of m/z 184. 0740 from fraction C indicating the presence of phosphocholines, Figure S2: Top: Ion chromatogram of m/z 496.3396 from fraction C showing the presence of LysoPC. Bottom: Base peak intensity chromatogram from the UHPLC-HR-MS/MS analysis of fraction C using positive electrospray, Figure S3: Top: Low energy mass spectrum of 1-Palmiotyl-sn-glycero-3-phosphocholine from fraction C. Bottom: High energy mass spectrum of 1-Palmiotyl-sn-glycero-3-phosphocholine showing fragments and mass deviations corresponding with the commercial standard, Figure S4: Top: Low energy mass spectrum of pheophorbide A from fraction D. Bottom: High energy mass spectrum of pheophorbide A showing fragments and mass deviations corresponding with the theoretical fragmentation of the database hit, Figure S5: Top: Low energy mass spectrum of hydroxypheophorbide A from fraction D. Bottom: High energy mass spectrum of hydroxypheophorbide A showing fragments and mass deviations corresponding with the theoretical fragmentation of the database hit, Table S1: The most abundant lysophosphatidylcholines in fraction C. The type indicates number of carbons and double bonds in the fatty acid moiety of the respective LysoPCs, and the retention times are given in minutes.
