# Supplementary material for: Lysophosphatidylcholines and Chlorophyll-Derived Molecules from the Diatom Cylindrotheca closterium with Anti-Inflammatory Activity
Source: Mar Drugs. 2020 Mar 17;18(3):166. doi: 10.3390/md18030166 (PMC7143213; doi:10.3390/md18030166)
Supplement: Supplementary file 1 [file marinedrugs-18-00166-s001.pdf]

Phosphocholines and chlorophyll derived molecules from the diatom *Cylindrotheca closterium* with anti-inflammatory activity

Chiara Lauritano [1,*], Kirsti Helland [2], Gennaro Riccio [1], Jeanette H. Andersen [2], Adrianna Ianora [1] and Espen Hansen [2]

[1] Department of Marine Biotechnology, Stazione Zoologica Anton Dohrn, CAP80121, Naples, Italy; chiara.lauritano@szn.it (C.L.); gennaro.riccio@szn.it (G.R.); adrianna.ianora@szn.it (A.I.)
[2] Marbio, UiT – The Arctic University of Norway, Breivika N-9037 Tromsø, Norway; kirsti.helland@uit.no (K.H.); jeanette.h.andersen@uit.no (J.H.A.); espen.hansen@uit.no (E.H.)
* Correspondence: chiara.lauritano@szn.it; Tel.: +39 081-5833-221

Supplementary information

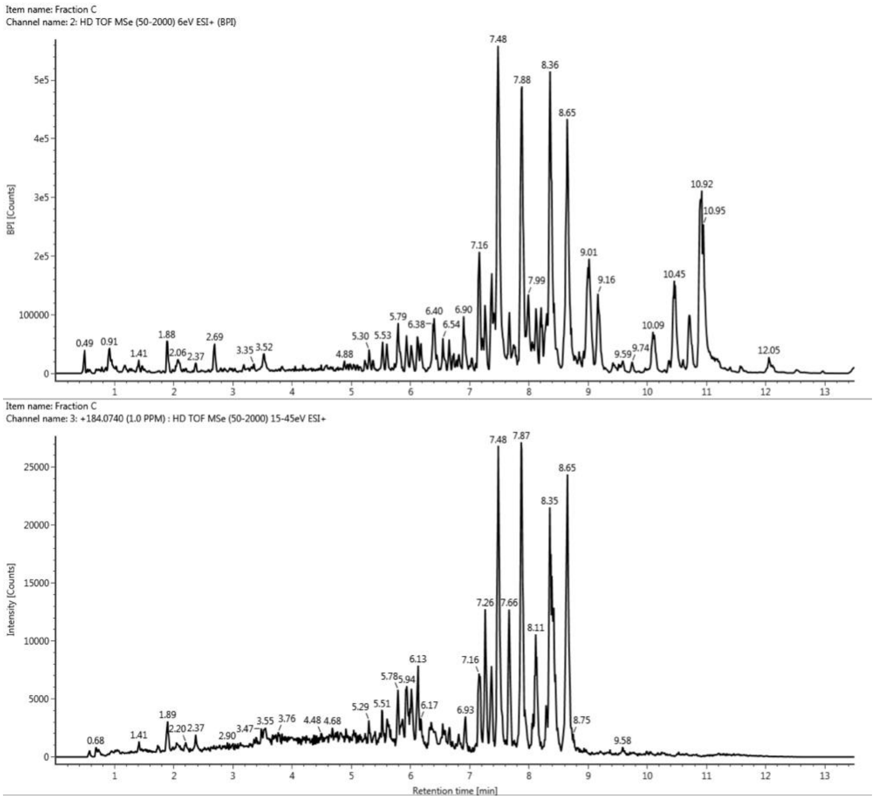

Figure S1. Top: Base peak intensity chromatogram from the UHPLC-HR-MS/MS analysis of fraction C using positive electrospray. Bottom: Ion chromatogram of m/z 184. 0740 from fraction C indicating the presence of phosphocholines.

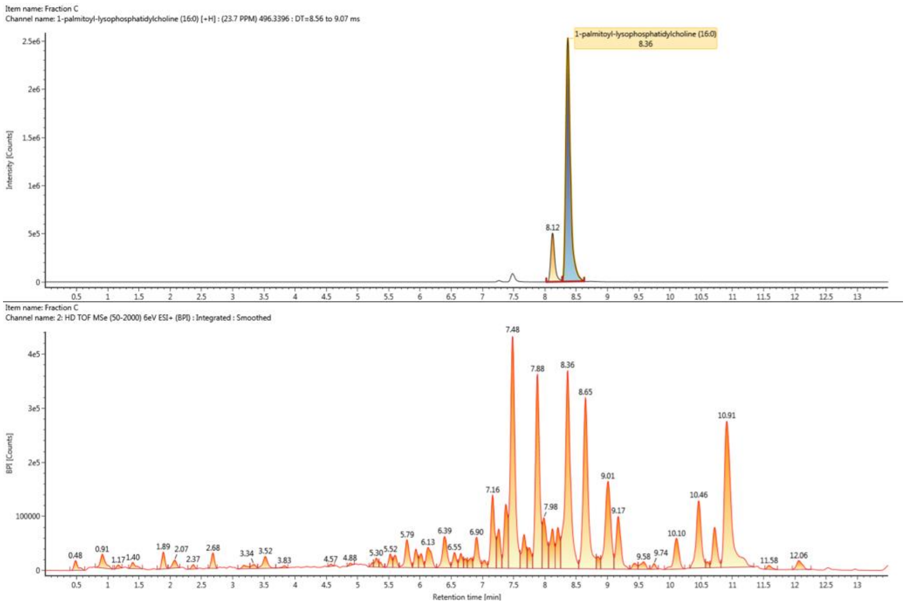

Figure S2. Top: Ion chromatogram of *m*/*z* 496.3396 from fraction C showing the presence of LysoPC. . Bottom: Base peak intensity chromatogram from the UHPLC-HR-MS/MS analysis of fraction C using positive electrospray.

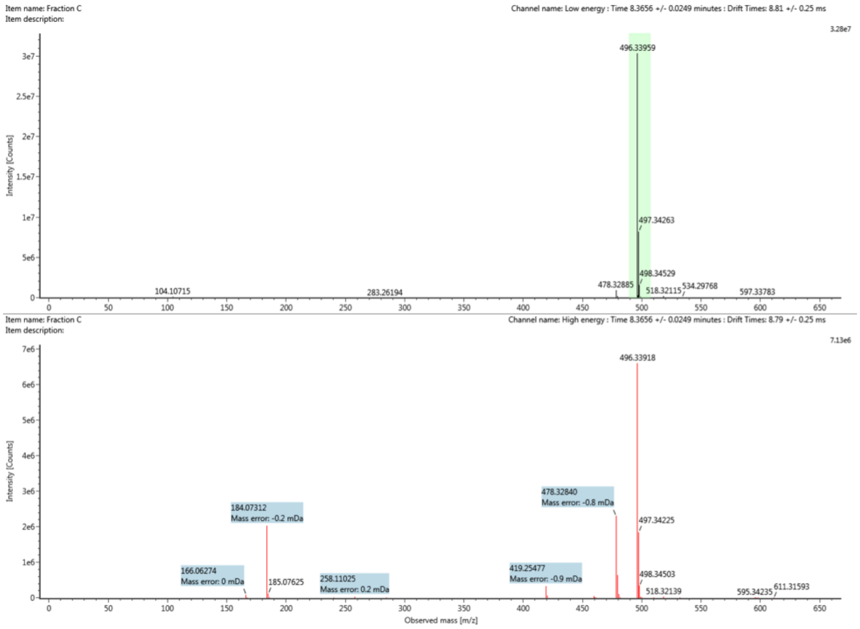

Figure S3. Top: Low energy mass spectrum of 1-Palmiotyl-sn-glycero-3-phosphocholine from fraction C. Bottom: High energy mass spectrum of 1-Palmiotyl-sn-glycero-3-phosphocholine showing fragments and mass deviations corresponding with the commercial standard.

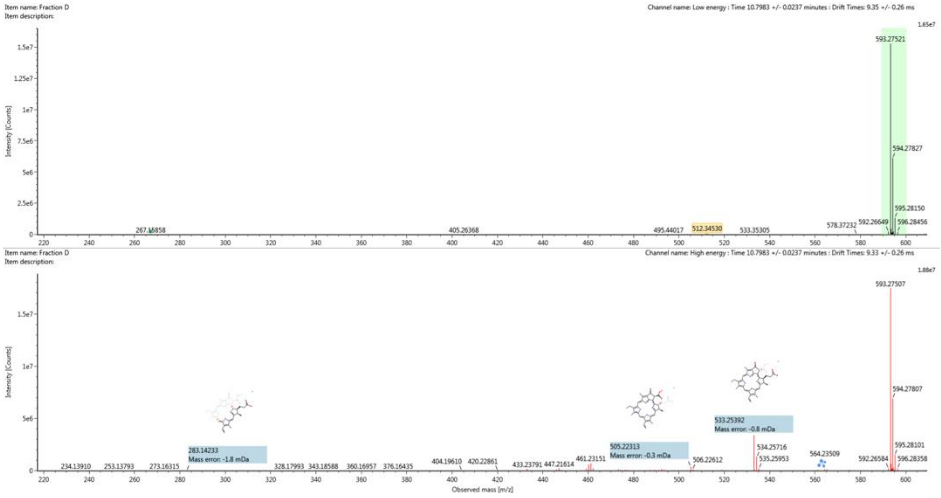

Figure S4. Top: Low energy mass spectrum of pheophorbide A from fraction D. Bottom: High energy mass spectrum of pheophorbide A showing fragments and mass deviations corresponding with the theoretical fragmentation of the database hit.

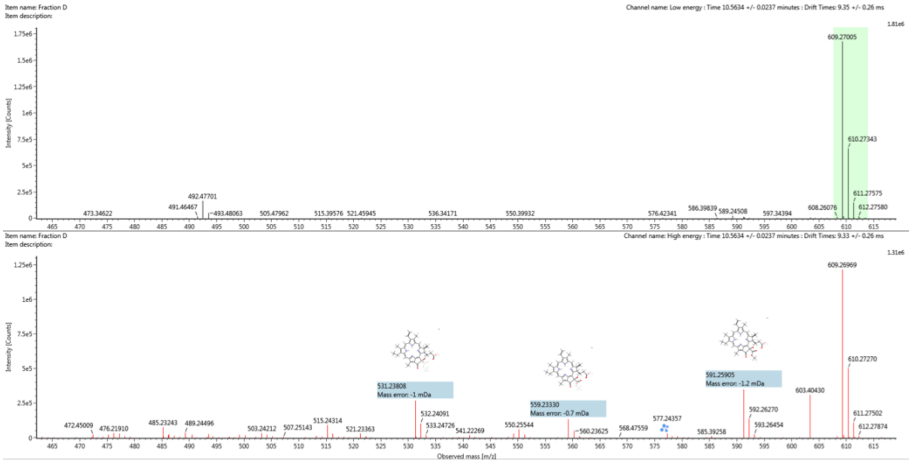

.

Figure S5. Top: Low energy mass spectrum of hydroxypheophorbide A from fraction D. Bottom: High energy mass spectrum of hydroxypheophorbide A showing fragments and mass deviations corresponding with the theoretical fragmentation of the database hit.

Table S1. The most abundant lysophosphatidylcholines in fraction C. The type indicates number of carbons and double bonds in the fatty acid moiety of the respective LysoPCs, and the retention times are given in minutes.

Number	Type	Observed mass (*m/z*)	Elemental composition	Retention time
1	14:0	468	$C_{22}H_{47}NO_7P$	7.16
2	18:3	518	$C_{26}H_{49}NO_7P$	7.36
3	16:1	494	$C_{24}H_{49}NO_7P$	7.48
4	18:2	520	$C_{26}H_{51}NO_7P$	7.88
5	16:0	496	$C_{24}H_{51}NO_7P$	8.36
6	18:1	522	$C_{26}H_{53}NO_7P$	8.65
